# Experience With Medical Treatment of Cesarean Scar Ectopic Pregnancy (CSEP) With Local Ultrasound-Guided Injection of Methotrexate

**DOI:** 10.3389/fmed.2020.564764

**Published:** 2020-11-03

**Authors:** Amandine Gerday, Amélie Lourtie, Céline Pirard, Pascale Laurent, Christine Wyns, Pascale Jadoul, Jean-Luc Squifflet, Marie-Madeleine Dolmans, Jean-Paul Van Gossum, Frank Hammer, Mathieu Luyckx

**Affiliations:** ^1^Department of Gynaecology and Andrology, Cliniques Universitaires Saint Luc, Woluwe-Saint-Lambert, Belgium; ^2^Institut de Recherche Clinique Pôle GYNE, Catholic University of Louvain, Brussels, Belgium; ^3^Institut de Recherche Clinique Pôle ANDRO, Catholic University of Louvain, Brussels, Belgium; ^4^Department of Gynaecology, Clinique Saint Jean, Brussels, Belgium; ^5^Department of Radiology, Cliniques Universitaire Saint Luc, Woluwe-Saint-Lambert, Belgium; ^6^Tumor Infiltrating Lymphocytes Group, Institut de Duve, Catholic University of Louvain, Brussels, Belgium

**Keywords:** Cesarean scar ectopic pregnancy (CSEP), medical treatment, fertility preservation, ultrasound, methotrexate (MTX)

## Abstract

**Objective:** Ectopic pregnancy within Cesarean section scars is a rare condition. Late diagnosis carries significant risk of bleeding with poor prognosis for survival. There is no consensus on the management of this type of pregnancy. Historically, our facility offered an intra-muscular injection of methotrexate that resulted in a significant failure rate and later need for surgery. We hypothesized that injecting methotrexate directly into the gestational sac would improve the success rate of the treatment.

**Patients and Methods:** This retrospective, uni-centric study examined nine patients aged between 33 and 42 years (mean age = 36.5 years) with Cesarean scar ectopic pregnancy (CSEP) between 2010 and 2018. CSEP was diagnosed by transvaginal ultrasound at a mean gestational age of 8w0/7. CSEP was treated under general anesthetic by ultrasound-guided methotrexate injection directly into the gestational sac. HCG levels and subsequent childbearing were monitored post-treatment.

**Results:** Half of the patients were asymptomatic at the time of diagnosis. All patients tolerated treatment well and all ectopic pregnancies were successfully removed. HCG levels returned to negative within 3 months without additional medical or surgical intervention. The post-treatment pregnancy rate was 50%.

**Discussions/Conclusions:** Our findings indicate that local ultrasound-guided injection of methotrexate into the gestational sac is a safe and effective therapeutic approach when performed by a trained team on a hemodynamically stable patient in the early stages of CSEP.

## Introduction

An ectopic pregnancy within the scar of a Cesarean section is a rare gynecological disorder. In patients with previous Cesarean sections, Cesarean scar ectopic pregnancy (CSEP) occurs in 1/1,800–1/2,216 of all pregnancies and it's 6% (1/16,6) of the all ectopics pregnancies (1, 2). The number of CSEP correlates with the number of Cesarean deliveries performed, and incidences have thus been rising in recent decades (1–3).

**Figure 1:**
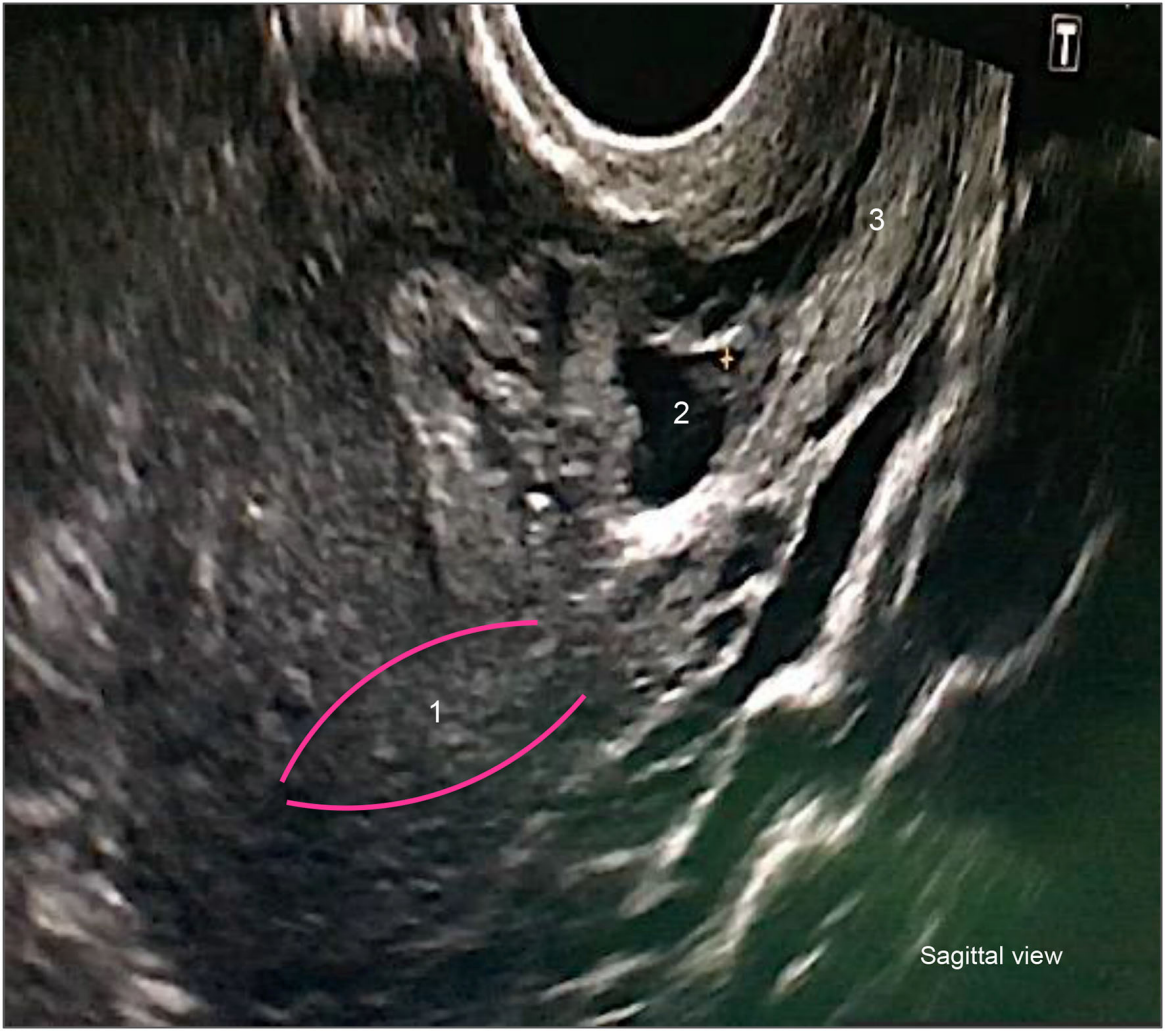
Cesarean scar pregnancy—Sonography. 1. Uterine cavity. 2. Cesarean scar pregnancy. 3.Cervix.

In 1978, Larsen et al. were the first to describe CSEP (4). In 1990, the first ultrasound diagnosis of CSEP was published (5). Today, a standard diagnosis of CSEP by transvaginal ultrasound requires the following criteria: presence of a gestational sac located at the site of a previous Cesarean section scar, decreased thickness of the myometrium located between the bladder and the site of the hysterotomy, the presence of peripheral hypervascularization around the gestational sac, and an empty uterine cavity and cervical canal (Figure 1).

The physiopathology of CSEP remains unclear. For example, Rotas et al. demonstrate that it remains unknown how the number of previous Cesareans impacts the risk of developing CSEP (1). Although potential risk factors for CSEP are underreported and poorly confirmed in the literature, several have been proposed: the presence of large Cesarean section scar dehiscence, the technique of closing the Cesarean wound at the hysterotomy site or a brief gap of time between a Cesarean delivery and a new pregnancy (6, 7), *in vitro* fertilization (IVF) techniques (8), and the difference between endometrial and myometrial tissue vascularization in the Cesarean scar niche and the rest of the endometrium (9).

CSEP carries a high risk of severe hemorrhage and death in later stages of pregnancy. Thus, termination is advised as soon as possible after diagnosis. Several cases of advanced CSEP that continued to progress into the second and third trimester have been described. The continuation of these pregnancies is mainly linked to missed diagnoses or to the patient's refusal to abort the pregnancy. Although some live births following CSEP have been described, these are rare and high risk to mother and child (10).

To date, consensus on the management of CSEP patients is lacking. Possible treatment strategies include medical or surgical intervention, or a combination of both. Most treatment strategies are designed to preserve the patient's fertility.

The most common medical treatment strategy is the injection of the anti-mitotic agent methotrexate. Methotrexate can be administered by systemic injection, intramuscular injection into the uterine wall (at 1 mg/kg body weight) or local injection directly into the gestational sac. The latter injection is guided by ultrasound or laparoscopy. Potassium chloride (KCl) may be injected instead of or in conjunction with methotrexate, particularly in the presence of a fetal heartbeat. Local injection of methotrexate with KCl is a more effective embryocide that also reduces the risk of hemorrhage (11). Osada et al. proposed using ethanol rather than methotrexate or KCl (12).

Surgical treatment involves the resection of the ectopic pregnancy by hysteroscopy, laparoscopy or laparotomy. Uterine artery embolization is often used prophylactically to reduce the risk of bleeding during other therapeutic procedures including curettages (13, 14) or hysteroscopies (15).

Treating CSEP may carry an increased risk of hemorrhage if the gestational sac is larger than 6 cm, the anterior wall is <0.2 cm thick, the systolic peak velocity is >70 cm/s, and the resistance index is <0.35 calculated from Doppler of peritrophoblastic blood vessels (16).

At our facility, standard diagnosis occurs by transvaginal ultrasound. Historically, the standard treatment consisted of intramuscular injection of methotrexate into the gluteal muscle (at 1 mg/kg body weight). Anecdotally, this treatment however was associated with a significant failure rate, which resulted in the patients requiring later surgical intervention. We hypothesize that changing the standard treatment protocol to injecting methotrexate directly into the gestational sac would improve the success rate of medically treating CSEP. However, we expect that highly trained personnel will be required to perform injections into small gestational sacs. Our retrospective, uni-centric exploratory study involved nine CSEP patients of which one experienced two CSEP.

## Method

In this uni-centric, retrospective study conducted between May 2010 and December 2018, we examined the effectiveness of local ultrasound-guided methotrexate injection into the gestational sac to treat Cesarean scar ectopic pregnancies (CSEP). We included data from nine patients and a total of ten procedures (one patient experienced CSEP twice). Our team reported this technique for the first time in 1997 (17).

CSEP was diagnosed by transvaginal ultrasound. Positive CSEP diagnosis required the following imaging criteria to be met (18):

- size of the gestational sac,
- presence of a yolk sac,
- presence of an embryo with or without cardiac activity,
- amount of peripheral vascularisation,
- remaining thickness compared to the bladder,
- differential diagnosis with cervical pregnancy.

Upon diagnosis, we offered patients to terminate the pregnancy by injecting methotrexate directly into the gestational sac located in the Cesarean section scar. The procedure was guided by ultrasound and was carried out by a member of the Medically-Assisted Reproduction (MAR) team who had been trained in managing similar procedures for oocyte retrieval for IVF. As CSEP is associated with risk of significant bleeding, we performed the injection in the operating theater under general anesthetic with a gynecological surgeon prepared to perform emergency hemostatic surgery. No patients experienced hemorrhage requiring surgery at the time of methotrexate injection. The methotrexate solution was prepared in the hospital pharmacy to one milligram per kilogram of body weight. After local disinfection, a puncture needle was introduced through the vagina into the gestational sac under ultrasound guidance. First, amniotic fluid was aspirated. Then, methotrexate was injected into the gestational sac and, if possible and present, into the fetus. If the gestational sac was too small to receive the entire methotrexate dose, any remaining methotrexate was injected intramuscularly into the uterine wall. The procedure concluded with a thorough check for any signs of active bleeding. All patients were observed clinically for 24 h.

Human chorionic gonadotropin (HCG) is a hormone that is released by the placenta during pregnancy and is used to test for pregnancy. To determine if the methotrexate injection terminated the CSEP, we conducted a first HCG level check and transvaginal ultrasound (on average) 7 days after the procedure. We carried out weekly blood tests for HCG until a negative pregnancy test was obtained. The patient used hormonal contraception throughout this period. Once HCG concentrations returned to negative levels (<5 IU/l), we assessed the Cesarean section scar for potential residual Cesarean scar niches by saline infusion sonography (SIS) or magnetic resonance imaging (MRI). This typically occurred within 3 to 6 months following the initial CSEP treatment. The choice of imaging depended on the patient's attending physician. If a residual niche was found, we proposed resecting the niche by laparoscopy prior to any further pregnancy (19, 20).

Here, we report ten cases of CSEP in nine patients who presented to our gynecology department between May 2010 and December 2018. One patient experienced two CSEP that occurred 1 year apart. All clinical, biological, radiological and therapeutic data were collected in an Excel spreadsheet. Some data were not available as several patients were referred from an external center for the management and treatment of CSEP. As these patients received their follow-up outside our institution, their data was only incompletely available.

## Results

Between May 2010 and December 2018, we treated nine patients ranging in age from 33 and 42 years (mean age = 36.5 years) for CSEP using a local ultrasound guided injection of methotrexate into the gestational sac (Table 1). The patients had experienced a maximum of three previous Cesarean sections (mean number of Cesarean sections before CSEP diagnosis = 1.6).

**Table 1:** Summary table of patients.

**Patient**	**Age**	**G P**	**Number of Cesarean**	**HCG J0**	**Age of the pregnancy**	**Time of standardization of the HCG (days)**	**Future pregnancy**
Patient 1	42	G4P1	1	NS	6w4/7	118	No pregnancy
Patient 2	36	G3P2	1	50,650	8w0/7	57	No pregnancy
Patient 3	33	G3P2	2	3,385	5w6/7	NS	No pregnancy
Patient 4	38	G7P4	2	59,742	7w5/7	75	Pregnancy - Cesarean
Patient 5 (first case)	38	G7P2	2	3,922	5w4/7	113	Ectopic pregnancy on the scar
Patient 5 (second case)	39	G8P2	2	5,097	8W0/7	56	Miscarriage
Patient 6	34	G3P1	1	9,840	5w4/7	NS	Pregnancy in progress
Patient 7	36	G5P3	1	25,224	11w0/7	118	No pregnancy
Patient 8	34	G2P1	1	14,930	5w 6/7	68	No pregnancy
Patient 9	34	G4P3	3	12,849	6W0/7	40	No pregnancy

*NS, non specified (monitoring the decrease in a secondary center)*.

In six out of 10 CSEP incidences, the patients reported no or very few symptoms at the time of diagnosis. This proportion of asymptomatic patients is comparable to the findings of Rotas et al. (1).

**Figure 2:**
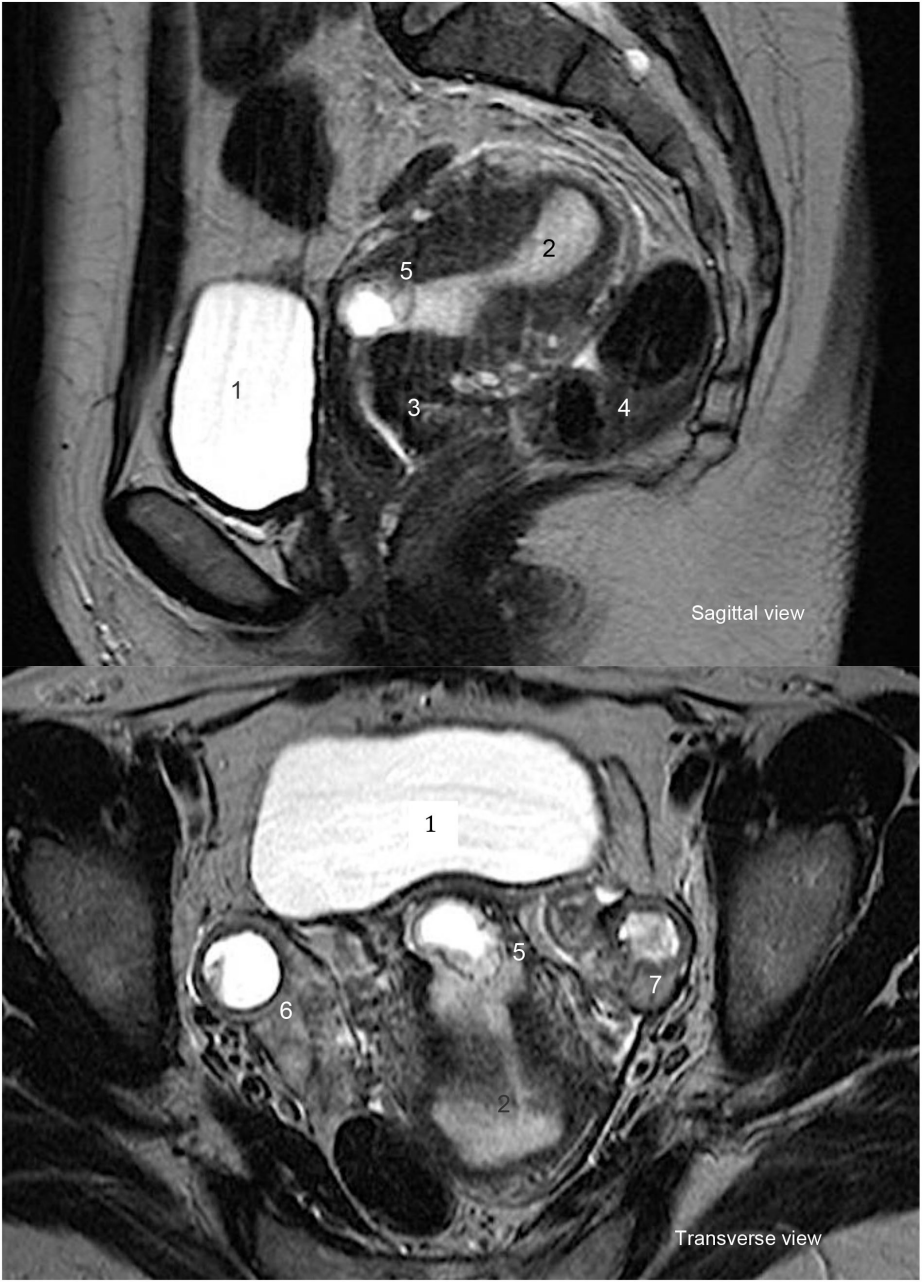
Cesarean scar pregnancy—MRI. 1. Bladder. 2. Uterine cavity. 3. Cervix. 4.Bowel. 5. Cesarean scar pregnancy. 6. Left ovary. 7. Right ovary.

**Figure 3:**
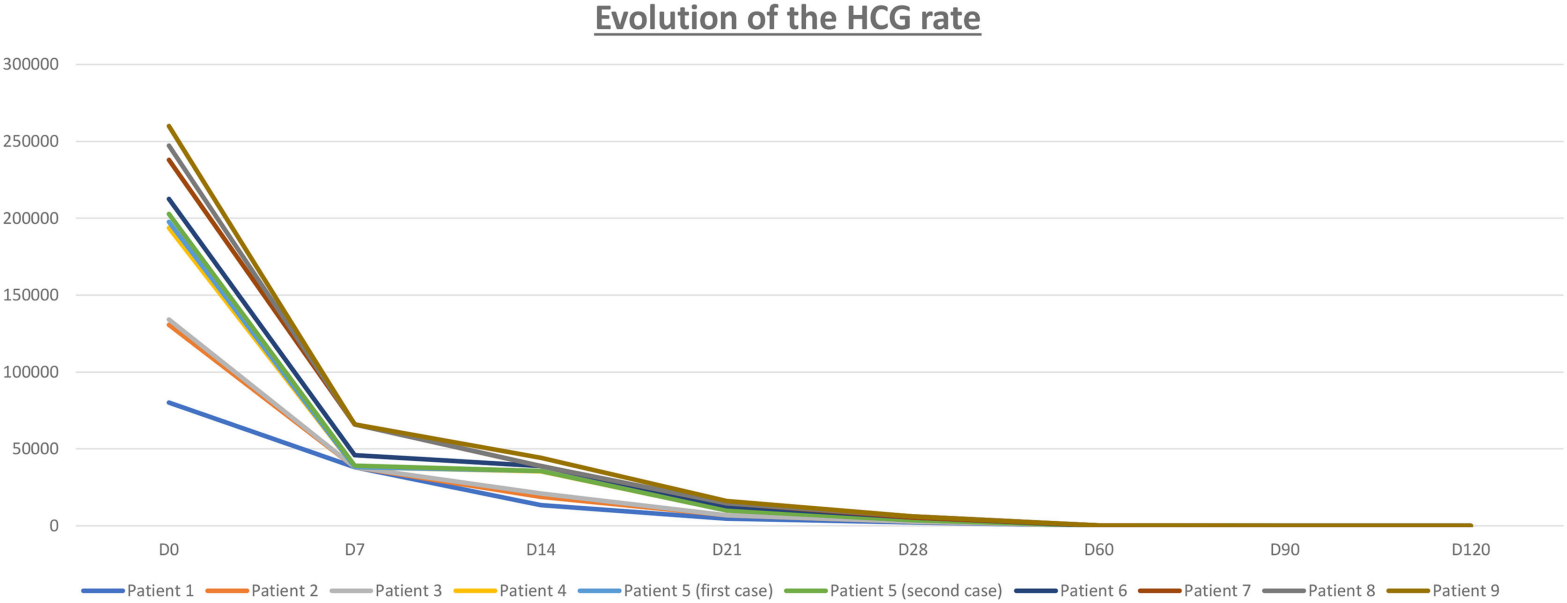
Evolution of the HCG rate (Human Chorionic Gonadotropin).

CSEP diagnosis occurred by transvaginal ultrasound and HCG blood tests for pregnancy. One of them received a pelvic MRI scan in addition (Figure 2). At diagnosis, the gestational sacs measured between 9 and 33 mm (mean = 18.5 mm). The mean gestational age at diagnosis was 8w0/7. In 75% of cases, a measurable fetal echo was identified with fetal cardiac activity in 80%. On the day of the methotrexate injection, the mean HCG level was 20112.8 UI/l (Figure 3).

No major complications were observed and no additional surgical intervention was required during or after the local ultrasound-guided methotrexate injection. As blood loss was minimal (<50 cc), no blood transfusions were required and none of the patients needed admission to intensive care.

Within 1 month following the methotrexate injection, three patients (patients 2, 8, and 9) were re-admitted for pain and/or bleeding. Patients 2 and 8 were clinically monitored without any additional treatment. Patient 9 required embolization (Embozene 400 and 700 μm) of the uterine arteries to stop the bleeding on day 24 after the methotrexate injection. To correct anemia, the patient was transfused one unit of blood before embolization. The patient progressed well clinically and HCG levels became negative within 18 days of embolization.

We monitored the serum HCG levels of all patients until they returned to negative (<5 IU/l), indicating the successful termination of pregnancy (Figure 3). Two weeks following methotrexate injection, HCG levels measured 5,351.3 IU on average (assessed between Days 10 and 20), which represents a decrease of more than 75% compared to the initial HCG level (average: 20112.8 IU/l). Negative HCG levels were observed within an average of 91.9 days following the methotrexate injection (Figure 3).

We assessed the Cesarean niche of all patients after treatment to monitor for possible dehiscence, potential CSEP recurrence and/or indication for Cesarean section scar resection. The Cesarean niche was assessed by pelvic MRI in seven patients, SIS ultrasound in one patient and transvaginal ultrasound in one patient. The choice of imaging technique depended on the patient's attending physician. Patient 5 alone required laparoscopic repair of the Cesarean section scar. This patient had a history of two CSEPs.

Of the nine patients treated, four became pregnant after the methotrexate injection for CSEP. The progress of all patients until today is chronologically detailed below (in Table 1):

- *Patient 1*: One year after methotrexate injection for CSEP, Patient 1 was diagnosed with breast cancer and ceased trying to fall pregnant again.
- *Patient 2*: After treatment, Patient 2 started oral contraception. She underwent bilateral tubal ligation within 3 years of treatment for CSEP.
- *Patient 3*: This patient underwent follow-up in a different medical center; thus, her follow-up data is incomplete. However, on D8 post-treatment her HCG had decreased by 33%. The patient presented in our department 2 years later for a simple consultation. She did not report any new pregnancy.
- *Patient 4*: Nineteen months after treatment for CSEP, Patient 4 delivered a child by Cesarean section. The pregnancy was unplanned.
- *Patient 5* (first case): Within 1 year after treatment with methotrexate injection, this patient experienced a second CSEP that was successfully treated by ultrasound-guided local injection of methotrexate into the gestational sac. After the second CSEP, the patient had a correction of the residual niche by laparoscopy.
- *Patient 5* (second case - after the laparoscopy): 1 year after treatment for the second CSEP, Patient 5 had an intrauterine pregnancy that miscarried spontaneously.
- *Patient 6*: Two years after CSEP treatment, this patient had a positive pregnancy test. Ongoing intra-uterine pregnancy was confirmed by ultrasound. It was a pregnancy obtained by IVF for unrelated reasons.
- *Patient 7*: To date, no new pregnancy occurred, although Patient 7 expressed a desire to become pregnant within the next few years.
- *Patient 8:* Nine months after the CSEP, the couple, who had been attempting IVF, requested the destruction of the remaining frozen embryos.
- *Patient 9:* This patient experienced an episode of acute vaginal bleeding on day 24 following the procedure. This bleeding was controlled by an embolization of the uterine arteries by our colleagues in interventional radiology. After this procedure, her HCG levels fell quickly. However, the patient had requested a conservative approach regarding her fertility and desired to avoid potentially radical surgery. After obtaining a negative HCG level, the patient was followed up in another medical center.

Only one of the nine patients expressed that she did not wish to become pregnant following the methotrexate injection for CSEP. To date, of the remaining eight patients four became pregnant, which represents a pregnancy rate of 50% after treatment for CSEP. It should be noted, however, that other causes of infertility (such as those experienced by Patients 1 and Patient 8) may impact the ability to conceive.

Patient 3 had a history of CSEP before she was admitted to our department. This is extremely rare. Only few cases have been described in the literature (21–23). Unfortunately, no information regarding her treatment during this episode is available. The two CSEPs happened 4 years apart. Following the methotrexate injection into the gestational sac, she reported no new pregnancy or CSEP.

Only Patient 5 presented with a second episode of CSEP. The two CSEPs occurred a year apart. Three months after the first CSEP, an MRI revealed a niche in the Cesarean scar, even though HCG levels had normalized by this time. The 6-month MRI showed no remaining niche and the patient was allowed to conceive. A second CSEP followed. The patient accepted termination of the CSEP by the same ultrasound-guided injection of methotrexate into the gestational sac. Six months after the second methotrexate injection, we discovered a dehiscence of the Cesarean section scar by MRI. The dehiscence and residual niche were corrected by laparoscopic resection. It is important to offer surgical correction of the cesarean scar if it has a niche in order to avoid the risk of CSEP.

## Discussion

CSEP is a rare condition that was first described in 1978. Recently, the incidence is rising as numbers of deliveries by Cesarean section also rise (2). Several risk factor for CSEP have been proposed but poorly verified (24). Proposed risk factors include the number of prior Cesarean sections, myomectomies, vacuum aspiration or curettage, and the use of IVF techniques using embryo transfer. We note that the review by Rotas et al. found no significant link between the number of prior Cesarean sections and the risk of developing CSEP (1).

Diagnosing CSEP is difficult because CSEP resembles a cervical pregnancy or miscarriage (with a cervical localization) that is being expelled. Commonly, transvaginal ultrasound is the best method to accurately diagnose CSEP. All CSEP presented in this report were diagnosed by transvaginal ultrasound.

Historically, our team treated CSEP by injecting methotrexate intramuscularly. However, anecdotally we observed a significant failure rate with this approach. Treatments had to be repeated or surgical interventions became necessary. We decided to attempt improving the success rate of treating CSEP, which led to the proposed ultrasound-guided local injection of methotrexate (17).

The methotrexate injection was performed by trained IVF gynecologists who were experienced in retrieving oocytes in a procedure that closely resembles methotrexate injection. Here, we presented the effectiveness of treating CSEP by injecting methotrexate directly into the gestational sac. With this technique, we observed very low complication rates and good preservation of fertility (as demonstrated by a pregnancy rate of 50%). Similar observations were found in the literature (25, 26). We hypothesize that injecting methotrexate directly into the gestational sac leads to improved effectiveness of treatment because a higher dose of methotrexate is delivered directly to the CSEP.

While MRI could assist diagnosis, it was more useful after methotrexate treatment for CSEP to assess the status of the Cesarean section scar. In case of dehiscence, as with Patient 5, surgical treatment is recommended (19, 20). Instead of or in addition to MRI, SIS ultrasound may be used to assess the Cesarean section scar after methotrexate treatment. In the absence of standard guidelines, each team offered medical and/or surgical treatment based on its experience. We observed no complications with either diagnostic tool.

Our study is limited because it is retrospective in nature, has a small number of patients and observed variations in the decreases of HCG levels. We recommend this procedure only for early cases of CSEP and hemodynamically stable patients. More advanced cases of CSEP (after 10 weeks) were surgically treated by laparoscopy or by laparotomy.

Injecting methotrexate directly into the gestational sac requires specialized centers with gynecologists who are familiar with retrieving oocytes by a similar MAR technique. The gynecologist should be assisted by a surgical team with experience in managing CSEP and its risks of hemorrhagic complications. Our study observed 90% success rate in our limited cohort (only one patient had to undergo a complementary treatment). High success rates are also described in the literature - particularly in Cheung's review, which reported a success rate of 73.9% for first-line single injections of methotrexate directly into the gestational sac (27).

Treating CSEP by locally injecting methotrexate enabled the patient's fertility to be maintained. We showed a post-treatment pregnancy rate of 50%. Monitoring the decrease in HCG levels to negative is essential. While HCG levels decrease, the patient may use effective oral contraception. After treating CSEP, we recommend that an evaluation of the scar is performed to assess whether a Cesarean niche remains that requires surgical repair before a further pregnancy is pursued. MRI or ultrasound-SIS may be performed to accurately assess the residual anterior myometrium and the size of any remaining Cesarean niche. To avoid over-estimating the size of the Cesarean niche, radiological examination should not be performed earlier than first 3–6 months following the methotrexate injection or until HCG levels are negative. If the patient conceives again after CSEP treatment, delivery routes should be discussed with the patient. Delivery options should account for the patient's obstetric, and especially CSEP, history. Generally, a Cesarean delivery is recommended (28).

## Data Availability Statement

The raw data supporting the conclusions of this article will be made available by the authors, without undue reservation.

## Ethics Statement

Ethical review and approval was not required for the study on human participants in accordance with the local legislation and institutional requirements. Written informed consent for participation was not required for this study in accordance with the national legislation and the institutional requirements.

## Author Contributions

AG, AL, PJ, J-LS, M-MD, J-PV, and ML: gynecologist/surgeon. CP, PL, and CW: gynecologist/IVF. FH: radiologist/interventional radiology. All authors contributed to the article and approved the submitted version.

## Conflict of Interest

The authors declare that the research was conducted in the absence of any commercial or financial relationships that could be construed as a potential conflict of interest.

## Abbreviations

CSEP: Cesarean scar ectopic pregnancy
HCG: Human Chorionic Gonadotropin
IVF: *in vitro* Fertilization
KCl: Potassium Chloride
MAR: Medically Assisted Reproduction
MRI: Magnetic Resonance Imaging
SIS: Saline Infusion Sonography.
